# COVID-19 as a Crisis of Confinement: What We Can Learn From the Lived Experiences of People With Intellectual Disabilities in Care Institutions

**DOI:** 10.1177/12063312231159232

**Published:** 2023-03-18

**Authors:** Adrienne de Ruiter, Alistair Niemeijer, Pieter Dronkers, Carlo Leget, Sara Dekking

**Affiliations:** 1 University of Humanistic Studies, Utrecht, The Netherlands

**Keywords:** care institutions, confinement, COVID-19, home, intellectual disability, vulnerable space

## Abstract

While the COVID-19 crisis has affected people all around the world, it has not affected everyone in the same way. Besides glaring international differences, disparities in personal and situational factors have resulted in strikingly dissimilar effects even on people within the same country. Special attention is required in this regard for people with intellectual disabilities (ID) who are vulnerable to marginalization and precarization during crises as concerns over safety and public health are likely to trump consideration for inclusion and care. This article explores the lived experiences during the pandemic of people with ID living in care institutions in the Netherlands. Particular attention is paid to the challenges involved in living through periods of confinement and separation in what may be called “vulnerable spaces.” Drawing from interviews with individuals with a mild ID who have been restricted in seeing family and friends through the closed access of group homes to visits from outsiders, as well as interviews with their relatives and support workers, the article considers the ways in which stakeholders have responded to these spatial policies and negotiated the meaning of living space in times of crisis.

## Introduction

It is sometimes said that home is where the heart is. The restricted access to care homes during the COVID-19 pandemic illustrates that this does not always ring true. Everyday life drastically changed for residents of care institutions during the COVID-19 crisis as their home was temporarily closed off from visits by family and friends and the outside world more generally. This article examines the ways in which individuals living in group homes for people with (mild) ID experienced these restrictive spatial policies and negotiated the meaning of domestic space during the pandemic in the Netherlands.

The measures that were imposed by the Dutch government and care organizations to reduce the risk of contagion had a severe impact on people with ID living in group homes, particularly in the early phases of the crisis.^1^ Daytime activities outside the home were canceled and a strict visiting regime was introduced, which only allowed clients to receive one visitor for 1 hr a day and only when this was considered essential to their well-being. In practice, this meant that many were not allowed to see family and friends for months. In several institutions, clients were confined to the home or compound during the first weeks of the crisis. In addition, rules that set out that clients and support workers should keep 1.5-m distance led to an increased sense of isolation. While clients had to maintain physical distance from each other, safety measures also required that they all went into quarantine if one or more of them developed symptoms that could point to a coronavirus infection. Clients living in group homes were thus exposed to a dualistic regime of confinement that categorized them as belonging to a distinctively vulnerable space that justified restrictive measures to keep them from engaging in contact with outsiders, yet simultaneously ruled out physical closeness between people on the inside. In other words, clients were subjected to a double bind of risk containment that blurred the lines between home and institution and private and public.

Our analysis illustrates that the prioritization of the group home as a vulnerable space where restrictive measures were imposed to keep residents safe from the coronavirus tended to undermine the group home as a domestic space. Intrusive measures to lower the risk of contamination impinged upon central values that allow people to feel secure, comfortable, and free in their living space, which support a sense of experiencing one’s domestic environment as (a) home.

The first part of the article considers literature on the meaning of home in general and group homes as domestic and vulnerable spaces in particular. The second part discusses the methods used for this study. The third part presents our findings, based on insights from the interviews conducted with individuals with mild ID living in group homes, their relatives, and support workers. This part examines the meaning that three central markers of the home—safety, comfort, and control—took on during the crisis for clients living in group homes. The final part sets out the various dimensions of vulnerability that these restrictive spatial policies expose, which illuminate how group homes constitute vulnerable spaces in a multiple sense.

## Group Homes as Domestic and Vulnerable Spaces

Scholarship on the meaning of home has long noted the tension between ideal-typical representations of the home as a space of belonging, comfort, and joy and realities that dispel the safe and secure image of domesticity that speaks from these often overly romantic depictions (Annison, 2000; Goldsack, 1999; Mallett, 2004). Back in 1903, Charlotte Perkins Gilman (2002) already described the juxtaposition between home as a place of “rest, peace, quiet, comfort, health [and] personal expression” (p. 3) and the home as a potential source of repression, particularly for women. Feminist writers have taken up this point and emphasized the constraining dimensions of the home as a place of captivity, isolation, and violence (Goldsack, 1999; Warrington, 2002). This shows that the centrality of the home for personal well-being, whether as a positive or negative influence, is widely acknowledged. In an insightful analysis of the meaning of home for people with physical disability, Rob Imrie (2004) observes that the home is “a context for social and mental well-being” (p. 746). In a similar vein, Fereshteh Lewin (2001) highlights how the home is a place where people can experience social, psychological, and cultural security.

Studies analyzing the meaning of home for people with ID in care institutions have compared and contrasted home experiences of individuals living with their family and in group homes. Early research in this field found that positive experiences and feelings of homeliness were related to a sense of self-direction and the ability to move around freely (Reiter & Bendov, 1996, p. 109). A more recent study confirms that quality of care in group homes links to opportunities for autonomy, which offer residents a sense of self-determination, and community participation, which enables social inclusion (Shipton & Lashewicz, 2017). These studies suggest that factors related to independence and control play a central role in the way in which people with ID experience their domestic environment.

Scholars have noted that domestic settings in group homes are often “highly regulated with conduct rules concerning curfews, visitations, chores, and pets” (Reiter & Bendov, 1996). These regulations generally seek to allow for freedoms, as these are important for the development and well-being of clients (MacLeod Budge & Wels, 2016). Yet, restriction and confinement are always a possibility, especially if residents expose problem behaviors. Claire Spivakovsky (2017) thus casts care institutions for people with ID as potential sites of confinement, noting how restrictive measures in this institutional setting often revolve around striking a “tenuous balance between welfare and security” (p. 368).

Confinement, punishment, and social control in the setting of group homes for people with ID revolve around tensions between care and control, and risks and needs. These tensions arise from the fact that group homes seek to offer clients the freedom to explore and extend their autonomy but also require the imposition of restraints, particularly when clients are judged to form a risk to themselves or others: “[G]roup homes are complex, *dualistic* sites of confinement, *and* regulated, fostered independence” (Spivakovsky, 2017, p. 380).

The dualistic nature of group homes for people with ID is also reflected in the fact that this setting simultaneously constitutes a domestic space and a space of caregiving. Residential care for people with ID generally involves costly, structured care services by trained (multidisciplinary) staff. It is a permanent form of care, which is interwoven with daily life where the emphasis lies on supporting and attending to the needs and (remaining) capabilities of the client. Research suggests that in domestic settings that double as a space of caregiving, clear lines between private and social space are often difficult to draw (Dyck et al., 2005). Clients living in group homes have limited privacy, as one of the structural features of residential care is living together with other people (who one did not choose to live with). As a consequence, clients do not have many options with regard to creating “zones of intimacy” for themselves other than resorting to their private bedrooms (Hauge & Heggen, 2008).

Available literature thus highlights tensions that mark the meaning of the home in general and the group home for people with ID in particular as a space of security, comfort, and freedom, as well as regulation, confinement, and repression. In light of the restrictive measures implemented in the domestic sphere for residents of care institutions during the pandemic, it is to be expected that residing in a group home takes on new meanings for clients as their living arrangement is cast as space of unique vulnerability requiring special restrictions. It is, therefore, important to consider the lived experiences of individuals with ID who have faced severe limitations in their everyday lives through the closure of their home. This analysis adds to broader scholarship on the relation between disability and vulnerability (Beckett, 2006; Scully, 2014) and on the various ways in which public (health) crises can heighten vulnerabilities of disabled persons (Goggin & Ellis, 2020; Riddle, 2021).

## Exploring Experiences With the Group Home During the Pandemic

The underpinning of this article involves an epistemological stance that a better understanding of living through confinement can be achieved by considering the lived experiences of those concerned. A qualitative approach was, therefore, chosen, consisting of semi-structured interviews, as this method is particularly useful for understanding meanings and experiences and for unpacking sensitive or complex issues in long-term care, such as views and feelings regarding confinement (Mays & Pope, 2000; Zwijsen et al., 2012). Individual semi-structured interviews were held with eight clients with a mild ID, five support workers, and three relatives (*N* = 16).

### Participant Selection

Participants were purposely selected within two care organizations through a combination of open calls and direct requests for participation. Two of the eight clients we spoke to turned out not to reside in a group home, but to live independently receiving ambulant care. A citation from one of these participants is included about feeling like a patient when support workers in protective gear paid a visit at home. The living arrangement of one client also differed from that of other participants. This client lives in a separate apartment on a compound for people with ID where shared communal spaces were closed off during the first weeks of the pandemic. Several of the participating clients were involved in client representation or provide experiential advice on living with (mild) ID to organizations, researchers, and other interested parties. Each of the clients had a mild ID, and some had additional developmental disabilities, such as autism.

The five support workers that were interviewed all provided assistance on group homes, during daytime activities, and/or in other supportive roles. Support workers were selected through a call disseminated by one of the organizations. The first author contacted one support worker directly for an interview. The three relatives are all parents of a person with mild ID living in a group home. Relatives were recruited through a call distributed to the members of the relatives’ representation committee of one of the care organizations. One relative was asked to participate by one of the interviewed support workers.

### Data Collection

The interviews were conducted by the first author and focused on the lived experiences of stakeholders with the pandemic and resulting measures. Conversations focused on the impact of the crisis on their lives. Most of the experiences recounted by participants took place during the first wave (mid-March–late-September 2020), as the most radical change(s) occurred in that period. Specific impactful moments during the second wave (late-September 2020–late-March 2021) and third wave (late-March–late-October 2021), related to quarantine or vaccinations, also came up.

The full topic list was covered in some interviews, while in others specific points that were particularly pressing for participants (e.g., experiences with quarantine) were discussed at length. A flexible semi-structured format was used to meet the individual needs of participants with mild ID in terms of focus and shared control over the flow and content of the interview. Interviews with support workers and relatives considered the challenges of maintaining high standards of care and close contact during the crisis. Interviews were held online or in person following the preference of the participant and lasted in between 30 and 75 min. The data-collecting process ran from June to October 2021.

### Ethical Considerations

Approval for the study was granted by the ethics committee of the University of Humanistic Studies. A special informed consent form for clients was drawn up in collaboration with advisers with a mild ID from one of the participating organizations. In two cases, the protocol for spoken informed consent was followed to meet the specific needs of individual participants. All interviews were recorded and fully transcribed. In case of live interviews, audio recordings were made. For online interviews, teams were used. Transcripts were anonymized. Recordings were erased after transcripts had been checked for accuracy.

### Data Analysis

The transcripts were coded by the first author. This process started out from a list of sensitizing concepts, which laid the foundation for the analysis of our research data (Bowen, 2006, p. 14). Our sensitizing concepts included central themes to the study presented in this article (e.g., “home” and “(loss of a) sense of home”). Since this study is part of an overarching research project on the impact of the COVID-19 crisis and resulting measures on vulnerable groups, the list also included various codes related to vulnerability, responsibility, and needs (e.g., “physical forms of vulnerability,” “sense of own responsibility,” and “need for proximity”). The code list was complemented and refined on the basis of central themes that inductively emerged from the interview material. The interpretation of interview data was discussed with other authors to consider various possibilities of meaning and identify shared views. All authors were involved in providing feedback throughout the process.

Regarding the transferability of findings, we favor the notion of “petite generalizations” as the aim of this study is to create an understanding of a specific lived experience (i.e., confinement during the pandemic) (cf. Abma & Stake, 2001). Detailed information is provided about the experiences of participants, as balanced against the needs for guaranteeing their anonymity, to provide readers the opportunity to assess the applicability of conclusions drawn to other cases that share similar characteristics.

## Group Homes as Spaces of Safety, Comfort, and Control in Times of Crisis

The discussion in this article focuses on how restrictions implemented in care institutions for people with (mild) ID in response to the pandemic affected the experiences of clients, relatives, and care providers with the group home as a domestic setting. Safety, comfort, and control were chosen as guiding themes to focus the discussion because these notions are central to understandings of the meaning of home (Chapman & Hockey, 1999; Imrie, 2004; Lashewicz et al., 2021).

### Safety: Confinement as a Trigger for Fear and Frustration

At the outset of the crisis, strict measures were imposed on all residents of care homes to reduce the risk of contagion. The quick rise of cases in the Netherlands led to understandable concern and a strongly perceived need to take prudent and stringent action to keep clients safe. Policies regarding care homes for people with ID tended to opt for erring on the side of caution as extensive restrictions were put in place. These measures had a strong impact on the everyday life of clients. A young woman who lives in a group home for people with a mild ID summarizes the extensive changes in her life: “A lot changed for us all of a sudden. No daytime activities, no parents. The home was virtually locked and you could not just go somewhere.”

The measures imposed, while contributing to the physical safety of clients in seeking to keep the coronavirus out the door, led to a heightened sense of unsafety and an increase in unsafe situations. Notably, the prohibition to see family and friends, the ban on (unnecessary) physical contact with support workers and fellow clients, and the pressures arising through being locked in a house together with persons that one did not choose to live with and who respond in different ways to the ongoing crisis mounted psychological, social, and emotional tensions.

The restricted access of group homes during the pandemic resulted for many clients in a feeling of isolation. One participant expresses that she felt like she had to face the crisis alone: “I was not allowed to go to my parents anymore and my parents were not allowed to visit me. So that was really difficult. Because all of a sudden you are on your own.” Relatives oftentimes play an important role as an (emotional) support system for people with ID. The abrupt ban on seeing relatives and friends, therefore, had a destabilizing effect on some clients, which was deepened by the fact that support workers were limited in their ability to comfort them as they were in principle not allowed to get close. One support worker notes that clients sometimes need physical contact to feel safe and build a relation of trust with care providers, which was theoretically foreclosed by the requirement to keep 1.5-m distance.

Another support worker observes that the measures took away from clients aspects of their lives that are fundamentally important to them. While for some seeing their family, boyfriend or girlfriend, or friends is central, for others participating in society through work matters most. Restrictions that kept clients from engaging in for them indispensable forms of contact or activities frequently led to feelings of frustration or hopelessness. Combined with a loss of daily structure in times of general uncertainty and anguish, these feelings translated for some into an escalation of behavioral problems. One support worker explains how certain clients engaged in aggression and self-mutilation. She wondered whether the end still justifies the means in situations like these: “What is more unsafe? I believe that behavioral problems in those cases cause greater unsafety than the risk of corona.”

Behavioral problems as expressed in (auto-)aggressive conduct jeopardize first and foremost the safety of the person(s) concerned, but also raise tensions in the group home, affecting the extent to which the home is experienced as a safe environment. One support worker describes how the team had to let certain clients leave the group home for daytime activities to ensure the safety of the group, noting how “when the clients are together 24/7 they turn into little time bombs waiting to explode.” The group home can also come to feel as an unsafe space in an emotional sense through rising tensions. A support worker on a group for young women with mild ID explains how disputes and irritation soared as clients were not able to take a break from each other by leaving the house. Several clients recount how they retreated to their rooms as shared spaces turned out to be too crowded or the scene for confrontations. This affirms Hauge and Heggen’s (2008) point that in domestic environments in care institutions often few options for privacy are available other than recurring to one’s bedroom.

The group home setting can lead to a sense of unsafety not only through a rise in anxiety, tensions, and behavioral problems but also through the perception of risks involved in living together with persons who respond differently to the crisis and safety measures. Several clients tell that they live with people who do not (fully) understand the restrictions or easily forget about them. One participant expresses a sense of unease about interactions with other residents in the group home:

I make sure not to come close to anyone. I do not embrace anyone. I do not kiss anyone. I do not get close to anyone if it is not necessary, if it is not allowed. I keep my distance. [. . .] But my flatmates they do not really care about my health. They just go stand one meter away from someone. Or they touch you.

These experiences of clients, support workers, and relatives illustrate how restrictive measures at times produce both a heightened sense of unsafety and an increase in unsafe situations in the group home, while a lack of strict adherence to the rules may also jeopardize people’s sense of security and expose them to health risks. Living together with people that one did not choose to live with forms a challenge for experiencing the group home as a safe domestic space as interdependence leads to the imposition of rules that are experienced as too demanding by some and concerns about a lack of carefulness by fellow clients for others.

### Comfort: The Home as a Strange and Unfamiliar Place

The crisis measures also impact on people’s sense of feeling comfortable in the group home. A home ideally offers a place that is familiar and where one can feel at ease. The regulations compromised this sense of comfort and familiarity by turning the group home increasingly into a space of caregiving governed by safety measures. A support worker describes the changes that occurred:

We started to use face masks. We had to wear gloves. Everyone had to wash their hands more often. [. . .] At first we put posters everywhere with how to wash your hands and where you have to wear a face mask.

After an initial period, the team decided that change was needed to balance the characteristics of the group home as a domestic space and a care facility: “This does not feel like home anymore. I mean, I do not have this kind of notes in my house.”

The use of face masks in particular led to feelings of estrangement and evoked a sense of intrusion. Several clients state that they are not always able to recognize support workers wearing a face mask. Face masks can cause a sense of unfamiliarity and confusion for people with a (mild) ID. One person who was interviewed for the research speaks about feeling like a patient when support workers came dressed in protective gear: “This kind of thing I expect in an operating or recovery room. Not in my house. This is more the kind of thing I expect in a hospital.” The hygiene procedures for returning to the group home after clients were allowed to visit relatives also caused unease:

I had to announce when I would come back because no one was allowed in the corridors. I had to walk to my apartment. Then I first had to take a shower, put on other clothes, and only then was I allowed to see people at one and a half meter distance.

Comfort in the home does not only concern what is familiar, however. The testimony of clients suggests that experiencing one’s domestic space as an agreeable environment in part also hinges on opportunities for change and diversity. One client expresses displeasure about the fact that for a long period they always saw the same people as residents were not permitted to leave the group home, staff members were assigned to fixed locations, and relatives and friends were not allowed to visit. Another client exclaims that for months they had to sit in the same room with the same people: “In one room. The whole time! That really is not enjoyable.” Boredom and weariness arise in this study as important factors that may render the group home a place where people do not want to be. Especially if people are not allowed to leave their home, an otherwise pleasant and comfortable domestic environment can come to feel suffocating.

A further factor that is impacted by the restrictive measures concerns social contact and closeness. Quinn et al. (2016) note that young people with complex disabilities report positive experiences of home when their living arrangements offer them a sense of belonging and potential for connecting with others. Safety regulations affected the conditions under which clients could maintain contact with fellow residents. Initially, many care organizations told clients to keep 1.5-m distance from each other. A client observed that it was strange that residents were no longer allowed to come close to each other as they live together. A relative hints at the way in which this double bind deprived clients in the first period of the crisis of all close contact as they were no longer allowed to see people from the outside, but also required to stay away from each other on the inside.

Both clients and support workers mention that despite the restrictions, ways were found to maintain a sense of connection. Clients stayed in touch figuratively, for example, by sitting out on the balconies of their apartments where they could talk with each other from a distance. Support workers and clients in certain group homes also engaged in regular conversations where residents together shared their experiences. The sense of social and emotional confinement was also mitigated through the acquisition of new skills, which allowed clients to enter into contact with people on the outside, such as video-calling with relatives and friends. While clients expressed a sense of pride about the competencies gained, they note that speaking with people online is not the same as seeing them in person.

### Control: The Group Home as a Place of Restriction

An important element of the spatial policies implemented in group homes relates to control. Group homes for people with ID are generally designed as a place that seeks to strike a balance between freedom and regulation (Spivakovsky, 2017). The pandemic, however, swung the balance decidedly toward the side of regulation, resulting in severe restrictions which led to confinement and constraint. One client tellingly sums up this comprehensive sense of limitation by noting that in the first weeks of the pandemic “we were not allowed to do anything.”

During the pandemic, living in a care institution became a ground for being subjected to far-reaching restrictions, which stood in stark contrast to the fact that care in group homes for people with a mild ID seeks to create space and opportunities for clients to try out new things and thereby extend their abilities (MacLeod Budge & Wels, 2016). For clients who were close to acquiring the competencies needed to live independently, the swift transition toward greater confinement entailed a significant drawback. A relative recounts how her child went from traveling alone with public transport to work, family, and friends to being allowed to have only short walks on the compound under supervision: “Why is he not allowed to walk alone on the compound? That little bit of freedom. Everything is taken from him.”

The obligation to stay at home gave some clients the feeling that they were locked up. Experiences with quarantine in particular caused a strong sense of confinement. Several clients speak about feeling like a prisoner in their own room. One client recounts this experience in language that evokes a vivid sense of captivity:

You have no way to go. You are stuck between four walls. Sometimes the staff brings some food. [. . .] They put the plate on the ground and I could take the plate from the ground and then I went back into my apartment.

Restrictions also pertain to activities like doing groceries, which limit the control clients have over what they buy and how they wish to buy it. A client says that this curbs her independence: “A piece of your freedom is taken away from you. Usually, you do your own groceries and you decide for yourself when you want to get things and how to get them.” Having support workers buy things for clients entails a further limitation of privacy as clients rely on them to purchase what they need over a period of months. This adds an extra layer of supervision as persons with ID are often under legal wardship, which forecloses possibilities to order goods online and increases their dependency on opportunities to go to physical shops.

Clients respond to these limitations of their freedom in a multitude of ways. Some used the periods of seclusion to get ahead on tasks. One client explains that he tidied and cleaned his house. The strategy of putting his apartment in order might be seen as a way of regaining a sense of control within the domestic sphere. Another tactic that was employed by clients was to listen to music as a form of distraction. Music is said to have “transporting” qualities that can take people’s mind of the moment and bring attention to the auditive experience (Magill, 2001), thereby potentially mediating the immediate experience of physical confinement. On the contrary, clients engaged in acts of subverting the rules by trying to break them, in particular by secretly grouping together with other residents or trying to run off. These acts may be seen as recalcitrant behavior in response to stringent restrictions, which following the accounts of clients, relatives, and support workers in this study, is a common pattern in people with mild ID. Yet, these forms of conduct may also be considered markers of the central importance of experiencing social connection and indicate limits to the ability to cope with near complete deprivation of close contact.

For support workers, it was often stressful to note how their working environment turned from “open groups” to “closed groups” and their role shifted from supportive and encouraging support workers to restricting and controlling “police officials.” Recognizing the needs of their clients for some respite and perspective in this long-standing crisis, support workers recount that they sought ways to allow them a sense of freedom and control within the constraints of the general rules. The recasting of the group home as a vulnerable space regulated by extensive safety restrictions was often difficult to unite with what one support worker described as the central objective of turning the group home into “a home for residents where they feel safe, where they feel they belong, where they can be themselves, and where we look for ways to get the best out of you.”

## Concluding Remarks: The Plural Dimensions of Vulnerable Space

Our findings indicate that the pandemic and the resulting crisis measures have left a significant imprint on the experiences of clients, relatives, and care providers with the group home as a domestic setting. Restrictive measures have affected the sense of safety, comfort, and control, which constitute central markers of the home. Strict regulations encroached on the group home’s function as a domestic space reconfiguring it predominantly as a space for caregiving. As the caregiving aspect took center stage, particularly during the early phases of the pandemic, the nature of the care provided in group homes also changed, as the experiences of clients, relatives, and care providers testify. While care in group homes usually focuses on providing space for clients to develop their competencies by trying out new things and taking managed risks, the crisis foregrounded caution and restraint, foreclosing important opportunities for people with a (mild) ID to learn and develop (MacLeod Budge & Wels, 2016).

The turn to confinement exposed various dimensions of vulnerability in the lives of people with (mild) ID living in group homes. While the pandemic entailed a significant challenge to all, it placed this group in a special predicament as their living arrangement resulted in even greater upheaval of their lives. Clients, relatives, and support workers also note that dealing with the crisis can be especially difficult for people with (mild) ID as their disability limits their cognitive, emotional, and social resources to cope with (quick) changes. Restrictive measures in turn reduced the means to draw on a support system of family and friends or find distraction outside the group home and find comfort with support workers or fellow clients within the group home. Heightened vulnerability is thus reflected in both situational and dispositional factors. Situational factors expose people with (mild) ID in group homes to more pervasive interference with their lives and restrictions to ways to cope with the uncertainty, frustration, and grief these changes may cause. Dispositional factors include cognitive, emotional, and social limitations to the ability to process the drastic changes caused by the pandemic and ensuing crisis measures. While these situational and dispositional factors highlight the negative sides to vulnerability, shared experiences of vulnerability also offer opportunities to connect as talking about the challenges that clients (and support workers) face during the crisis led to a stronger sense of solidarity and understanding. Vulnerability can, therefore, contribute to the production of deeper forms of connection, which can strengthen the sense of belonging and feeling secure and comfortable in the group home (Quinn et al., 2016).

The understanding of vulnerable space that underpinned the justification for restrictions relied on a partial view of the vulnerability inherent in group homes as a space of care. Our analysis suggests that care institutions for people with ID constitute vulnerable spaces in the pandemic in a plural sense. At least three distinctive meanings of “vulnerable space” can be distinguished here: (1) A space may be labeled as vulnerable by relevant authorities, marking it off for special interventions to keep those belonging to it safe; (2) A space may be experienced as vulnerable by those inhabiting it, as their environment renders them (especially) susceptible to harm and suffering; (3) A space may provide a safe place for vulnerable encounters through which deeper forms of connection can be established between persons engaged in sharing their personal experiences.

The congregated nature of life in group homes and the inability to consistently maintain distance and uphold safety measures while providing necessary care to people with special needs exposed residents and support workers to heightened risk of infection. These factors designated group homes as a vulnerable space in the first sense, as listed above, requiring specific measures to keep residents safe from contagion. The measures taken to reduce this risk, however, heightened the psychological, emotional, social, and bodily vulnerability of residents as experiences of confinement and isolation led to distress, isolation, depression, and an increase in self-harming and aggressive behavior. Central elements to the sense of home—such as, safety, control, and freedom—were undermined through spatial policies of closure and confinement, which turned the group home into a vulnerable space in the second—experiential—sense in failing to respond adequately to the social, emotional, and physical needs of residents. As support workers and clients sought to mediate these factors by sharing experiences of vulnerability, a feeling of homeliness could in some cases be maintained or restored by tapping into the potential of vulnerable spaces—in the third sense—to create a sense of social connection.^2^

## Notes

1. The early phases of the crisis refer here to the first 3 months of the national crisis response in the Netherlands, which ran from mid-March until mid-June 2020. On March 17, 2020, limitations to daytime activities outside the group home were introduced. The closure of care institutions for visitors was announced on March 23, 2020. From June 1, 2020, all clients were guaranteed access to daytime activities again. From June 15, 2020, clients were allowed to receive visitors.

2. The finding that experiences of vulnerability can serve as a common ground that may help strengthen a sense of home offers a promising lead for future research. To fully tap into this potential of vulnerable space, it would be worthwhile to look into ways of fostering dialogue between residents and support workers—both within and across group homes. Paying close attention to the experiences of those concerned is a crucial first step in assessing what is needed to turn the group home into a safe space where vulnerability can be shared in supportive ways. Examining which factors bolster and impede the cultivation of generative vulnerable space can contribute to establishing conditions where adversity can be faced together from a place that provides safety, comfort, and control.
